# Moderate alcohol consumption on the risk of stroke in the Million Veteran Program

**DOI:** 10.1186/s12889-023-17377-x

**Published:** 2023-12-12

**Authors:** Rebecca J. Song, Martin G. Larson, Hugo J. Aparicio, J. Michael Gaziano, Peter Wilson, Kelly Cho, Ramachandran S. Vasan, Matthew P. Fox, Luc Djoussé, On Behalf Of the Million Veteran Program

**Affiliations:** 1 MAVERIC VA Boston Healthcare System, Lafayette City Center, 2 Avenue de Lafayette, Boston, MA 02111 USA; 2 Department of Biostatistics, Boston University School of Public Health, Boston, USA; 3 Department of Medicine, Boston University School of Medicine, Boston, USA; 4 Department of Neurology, Boston University School of Medicine, Boston, USA; 5 Division of Aging, Department of Medicine, Harvard Medical School, Boston, USA; 6 Atlanta VA Medical Center, Decatur, GA USA; 7 Emory University Schools of Medicine and Public Health, Atlanta, GA USA; 8 Section of Preventive Medicine, Boston University School of Medicine, Boston, USA; 9 Department of Epidemiology, Boston University School of Public Health, Boston, USA; 10 Department of Global Health, Boston University School of Public Health, Boston, USA; 11 Department of Nutrition, Harvard T.H. Chan School of Public Health, Boston, USA

**Keywords:** Alcohol consumption, Stroke, Cohort study, U.S. Veterans, Bias analysis

## Abstract

**Background:**

There is inconsistent evidence on the association of moderate alcohol consumption and stroke risk in the general population and is not well studied among U.S. Veterans. Furthermore, it is unclear whether primarily drinking beer, wine, or liquor is associated with a difference in stroke risk.

**Methods:**

The study included 185,323 Million Veteran Program participants who self-reported alcohol consumption on the Lifestyle Survey. Moderate consumption was defined as 1–2 drinks/day and beverage preference of beer, wine or liquor was defined if ≥ 50% of total drinks consumed were from a single type of beverage. Strokes were defined using ICD-9 and ICD-10 codes from the participants’ electronic health record.

**Results:**

The mean (sd) age of the sample was 64 (13) years and 11% were women. We observed 4,339 (94% ischemic; 6% hemorrhagic) strokes over a median follow-up of 5.2 years. In Cox models adjusted for age, sex, race, education, income, body mass index, smoking, exercise, diet, cholesterol, prevalent diabetes, prevalent hypertension, lipid-lowering medication, antihypertensive medication, and diabetes medication, moderate alcohol consumption (1–2 drinks/day) was associated with a 22% lower risk of total stroke compared with never drinking [Hazards ratio (HR) 95% confidence interval (CI): 0.78 (0.67, 0.92)]. When stratifying by stroke type, we observed a similar protective association with moderate consumption and ischemic stroke [HR (95% CI): 0.76 (0.65, 0.90)], but a non-statistically significant higher risk of hemorrhagic stroke [HR (95% CI): 1.29 (0.64, 2.61)]. We did not observe a difference in ischemic or hemorrhagic stroke risk among those who preferred beer, liquor or wine vs. no beverage preference. When stratifying by prior number of hospital visits (≤ 15, 16–33, 34–64, ≥ 65) as a proxy for health status, we observed attenuation of the protective association with greater number of visits [HR (95% CI): 0.87 (0.63, 1.19) for ≥ 65 visits vs. 0.80 (0.59, 1.08) for ≤ 15 visits].

**Conclusions:**

We observed a lower risk of ischemic stroke, but not hemorrhagic stroke with moderate alcohol consumption and did not observe substantial differences in risk by beverage preference among a sample of U.S. Veterans. Healthy user bias of moderate alcohol consumption may be driving some of the observed protective association.

**Supplementary Information:**

The online version contains supplementary material available at 10.1186/s12889-023-17377-x.

## Introduction

The observed association of lower cardiovascular disease risk with moderate alcohol consumption continues to be a debated topic. Previous studies have found a protective association of moderate alcohol consumption on cardiovascular disease (CVD) risk, compared with alcohol abstention or heavy drinking [1, 2]. A reduction in coronary artery disease risk has been more consistently observed with moderate alcohol consumption, but the association is less clear for stroke, which may indicate a difference in relations by CVD type [3, 4]. There is inconsistent evidence from observational studies in the general population where some have reported an 8–29% lower risk of stroke risk with moderate alcohol consumption [5–7], while others have observed no association [8–10]. Furthermore, the relation of alcohol intake with stroke risk among U.S. Veterans, who have lower health status [11, 12] and higher alcohol assumption [13], is not well known.

It is also unclear whether the type of alcohol consumed has an impact on stroke risk among U.S. Veterans. Previous studies have reported that red wine versus beer provides the greatest benefit in reducing the risk of CVD – largely attributed to the polyphenols found in red wine, which reduce platelet aggregation, increase high-density lipoprotein cholesterol and provide anti-inflammatory benefits [14]. However, a meta-analysis of studies assessing wine, beer, and spirit intake observed similar protective associations against CVD risk between those who drink moderate amounts of wine or beer compared with never drinking, and no protective association among those who drink spirits [15]. Lastly, selection bias, reverse causation, and residual confounding in observational studies are also possible explanations of the observed U-shaped relation of alcohol consumption and CVD risk, as noted in recent studies of alcohol consumption [16–18].

The primary aim of this study is to estimate the association of moderate alcohol consumption compared with never drinking on the risk of incident stroke using among U.S Veterans. The secondary aim is to assess whether, among moderate drinkers, beverage preference of wine, liquor, or beer has a different association on stroke risk compared to consuming a relatively even mixture of all types.

## Methods

### Study population

**Fig. 1:**
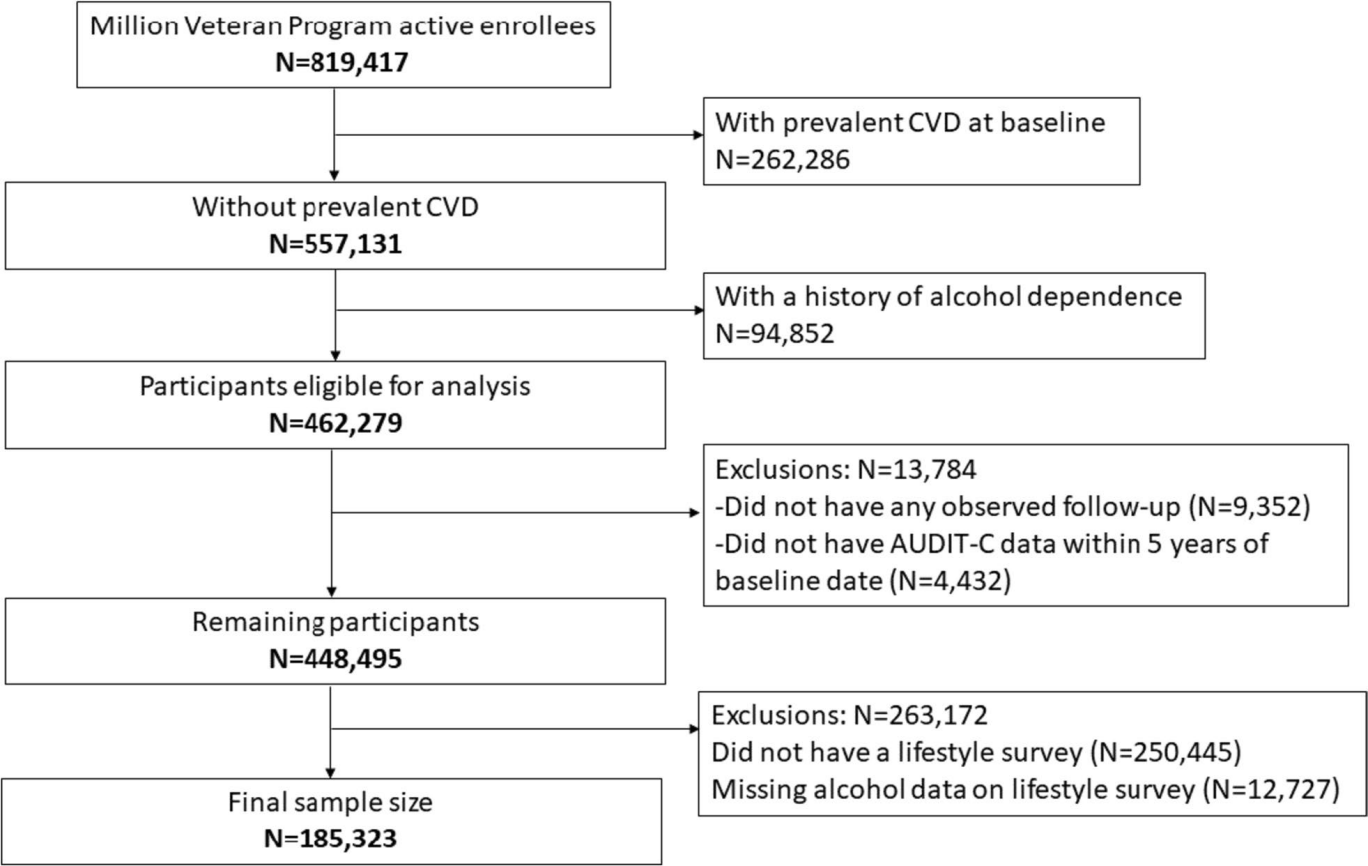
Inclusion and exclusion criteria of the final study sample of Million Veteran Program enrollees

We included participants enrolled in the Veterans Affairs Million Veteran Program (MVP), an ongoing prospective cohort study, which aims to study genetic and lifestyle risk factors of several diseases [19]. The Million Veteran Program began recruitment in 2011 and has enrolled 819,417 Veterans as of September 30, 2020. Enrollees who did not have a history of CVD or alcohol dependence before enrollment were eligible for inclusion (*N* = 462,279). We excluded individuals who did not have any observed follow-up (*N* = 9,352) or were missing alcohol consumption data in their EHR from five years prior to their enrollment date (*N* = 4,432). Lastly, we excluded individuals who did not have a Lifestyle Survey which includes self-reported information on alcohol consumption (*N* = 263,172), resulting in a final sample size of 185,323 participants (Fig. 1). Million Veteran Program participants provided informed consent to participate in the study. The Veterans Affairs Institutional Review Board (IRB) approved the study protocol and all methods were carried out in accordance with the VA IRB's regulations.

### Exposure

Alcohol consumption was self-reported on the Lifestyle Survey, which also asks about demographic characteristics and lifestyle behaviors, including physical activity and diet, using a food frequency questionnaire. All MVP participants are invited to complete a Lifestyle Survey once enrolled. The Lifestyle Survey asks participants to report their alcohol consumption status (never, former, or current), and the food frequency questionnaire specifically asks participants about their average consumption of beer (1 glass, bottle, can), wine (4 oz.), and liquor (1 drink or shot) over the past year. The possible response categories include: never or < 1/month, 1–3/month, 1/week, 2–4/week, 5–6/week, 1/day, 2–3/day, 4–5/day, and 6 + /day. For current drinkers, we converted each participant’s responses for beer, wine, and liquor intake to reflect drinks/day by taking the mean of the drink range in each response category and dividing it by the number of days. For example, a participant who selected 1/week would be consuming (1/7) or 0.14 drinks/day, and a participant who selected 2–3/day would be consuming 2.5 drinks/day. The responses for beer, wine, and liquor were summed to obtain the total drinks consumed daily. The final exposure categories were as follows: Never, Former, < 1 drink/day, 1–2 drinks/day, > 2–3 drinks/day, ≥ 3 drinks/day. We defined moderate consumption as the single category of 1–2 drinks/day. The primary analyses use the Lifestyle survey-defined alcohol consumption exposure.

To define beverage preference (of beer, wine, or liquor), we assigned preference to the type of beverage that provided ≥ 50% of the total drinks/day consumed among light and moderate drinkers (< 1 drink/day and 1–2 drinks/day categories). Participants were considered as having no beverage preference if a single beverage type did not account for ≥ 50% of the total consumption.

We also defined alcohol consumption using responses from the Alcohol Use Disorder Test-Concise (AUDIT-C) questionnaire, a screening tool for unhealthy alcohol consumption, which is regularly administered as part of clinical care in the Veterans Affairs healthcare system [20]. The AUDIT-C is stored in the EHR and was not restricted to those who only completed a Lifestyle Survey, and 99% of eligible participants in the current analysis had completed at least one AUDIT-C from the previous 5 years. We converted responses to the AUDIT-C questions that ask about alcohol consumption frequency and amount to obtain drinks/day among, restricted to those who did not report binge-drinking behavior, similarly to the survey-based conversion. Participants who answered “never” on the AUDIT-C question related to consumption frequency and had null or selected “never” on the questions about consumption amount were classified as never drinkers. The final exposure categories using the AUDIT-C measure were as follows: Never, < 1 drink/day, 1–2 drinks/day, > 2–3 drinks/day, and ≥ 3 drinks/day. Similarly, we defined moderate consumption using the single category of 1–2 drinks/day. We primarily use the AUDIT-C defined alcohol consumption exposure in sensitivity analyses.

### Outcome

The primary outcome was ischemic and hemorrhagic stroke defined as having one inpatient or two outpatient International Classification of Diseases (ICD) 9 and 10 codes for stroke from the EHR or defined using a previously validated phenotyping algorithm for ischemic stroke [21]. We used the following codes for ischemic stroke: 434.xx, 436.xx (ICD-9) and ICD-10 I63.xx, I64.xx or I69.3 (ICD-10); and the following codes for hemorrhagic stroke: 430.xx, 431.xx (ICD-9) and I60.xx, I61.xx (ICD-10). Follow up began at the enrollment date and continued until an incident stroke occurred or censored on their last hospital visit date (obtained from the EHR) or date of death.

### Covariates

We obtained demographic and baseline characteristics from the MVP data repository, which uses self-reported data from the survey and the EHR up to the enrollment date (baseline date). Demographic characteristics included date of birth (to calculate age at enrollment), sex, race, ethnicity, and income. Education, exercise frequency, diet quality (assessed using the Dietary approaches to Stop Hypertension [DASH] score [22, 23]) were obtained from the survey and were not available for participants who did not complete the survey. We obtained smoking status from the EHR Health Factors data, which contain responses from lifestyle-related questionnaires, including smoking status, administered during clinic visits. We obtained clinical covariates taken closest to the baseline date for total and high-density lipoprotein (HDL) cholesterol, as well as height and weight (to calculate body mass index (BMI)). Prevalent diabetes was defined as having either an ICD-9 code of 250.xx or the use of diabetes medication prior to the baseline date. Prevalent hypertension was defined as having an ICD-9 code of 401.xx or the use of antihypertensive medication. Lipid-lowering, diabetes, and antihypertensive medications were obtained from the outpatient pharmacy records.

### Statistical analysis

We compared baseline characteristics among the six Lifestyle Survey-defined alcohol consumption categories. We performed multiple imputation of 5 datasets using the fully conditional specification method to impute missing data for covariates. The proportion of missing data for each covariate ranged from 0.2–10.9%. We used Cox proportional hazards regression to estimate hazards ratios (HR) and 95% confidence intervals (CI)) to assess the association of alcohol consumption and incident stroke (composite ischemic and hemorrhagic), using never drinkers as the reference group, and also < 1 drink/day as the reference group. We reported the summarized estimates and standard errors of the 5 imputed datasets using PROC MIANALYZE in SAS 9.4. We first estimated age- and sex-adjusted hazards ratios (Model 1), and then multivariable-adjusted models (Model 2) additionally adjusting for race, education, income, BMI, smoking, exercise frequency, DASH score, prevalent diabetes, prevalent hypertension, lipid lowering medication use, diabetes medication use, antihypertensive medication use and total/HDL cholesterol ratio. We stratified models by age (≤ 40, 41–60, > 60 years) as an assessment of the presence of healthy survivor bias – individuals who might have been susceptible to alcohol-related injury at an earlier age would have been excluded from the study population and thus healthier individuals who may not be as susceptible to alcohol-related injury were included. We also stratified by sex, and stroke subtypes of ischemic and hemorrhagic stroke separately. Lastly, we stratified the analyses by categories of ≤ 15, 16–33, 34–64, ≥ 65 visits as a proxy for overall health to assess the possibility of reverse causation (i.e., those with poor health leads them to quit or reduce drinking).

For the secondary aim, we restricted our analyses to current light-moderate drinkers (< 1 and 1–2 drinks/day categories). We used Cox proportional hazards regression to estimate HRs and 95% CIs for the incidence of ischemic and hemorrhagic stroke, separately, among those who prefer beer, wine, or liquor compared to those who have no preference adjusting for the same covariates previously mentioned in Models 1 and 2.

We conducted a quantitative bias analysis to determine the potential impact of non-differential exposure misclassification of moderate vs. never alcohol consumption on the estimates of incident stroke using previously developed methods [24]. It is possible that moderate drinkers self-reported as never drinkers due to social desirability [25], or may have recently stopped drinking. Due to the prospective nature of this study, the misclassification was likely not related to incident stroke status, thus non-differential. We combined < 1 drink/day and 1–2 drinks/day as one moderate consumption category to create a binary exposure and made assumptions about the sensitivity and specificity of exposure in our observed data. We used a distribution of sensitivity between 0.84–0.95 and specificity between 0.94–0.99 because a previous validation study of self-reported alcohol reported lower sensitivity but high specificity [26]. We calculated an “adjusted” number of exposed/unexposed and disease/not diseased using 2 × 2 contingency tables. We then used the adjusted data to back-calculate the resulting positive predictive values (PPV) and negative predictive values (NPV), the probability of being correctly classified given the observed classification [27]. We simulated a new bias-adjusted binary alcohol exposure using 10,000 datasets drawing from a Bernoulli distribution using the PPV and NPV for the probability parameters and ran the Cox models using the bias-adjusted exposure. We summarized the distribution of 10,000 bias-adjusted hazards ratios using the 2.5 and 97.5 percentiles to produce a 95% simulation interval around the median estimate to capture the systematic error. We also incorporated random error by selecting a random normal deviate multiplied by the conventional standard error from the Cox models to capture the total (systematic + random) error. Additionally, we assessed the potential impact of differential exposure misclassification with respect to prevalent hypertension or diabetes. Those who have these conditions may be more likely to report as “never” drinkers when they may have been moderate consumers but quit due to their condition. We used a distribution of sensitivity among those with prevalent diabetes or hypertension between 0.82–0.95 and among those without these conditions between 0.90–0.95. For specificity, we used a distribution between 0.93–0.99.

We also examined whether our results may have been biased by selection due to those who have completed a Lifestyle Survey (*N* = 198,050) and those who did not (*N* = 250,445) and were thus excluded from the primary analysis. We compared baseline characteristics between those included and excluded from the primary analysis to determine whether those who chose to complete the survey differed with respect to alcohol consumption and covariates compared with those who did not complete the Lifestyle Survey. We also estimated the association of moderate alcohol consumption using the AUDIT-C defined alcohol consumption exposure, which was EHR-based and not restricted to those who completed a Lifestyle Survey, to assess whether the estimates for moderate alcohol consumption differed between those who were included and excluded from the primary analysis.

## Results

The baseline characteristics of 185,323 participants with a lifestyle survey are summarized in Table 1 by alcohol consumption category. Compared with never drinkers, moderate drinkers were slightly younger, had a lower proportion of women, non-White individuals, medication use, prevalent diabetes, and hypertension, and had higher educational attainment, income, exercise frequency, current smokers, and HDL cholesterol.

**Table 1:** Baseline characteristics among 185,323 Million Veteran Program participants who completed a lifestyle survey by alcohol consumption

**Characteristic**	**Never**	**Former**	**Current drinkers**			
			** < 1** **drink/day**	**1–2 drinks/day**	** > 2–3 drinks/day**	** ≥ 3 drinks/day**
	***N* = 16,847**	***N* = 65,349**	***N* = 74,346**	***N* = 13,883**	***N* = 8,678**	***N* = 6,220**
Age at enrollment, years	65.5 ± 12.7	64.4 ± 11.7	62.4 ± 13.3	65.6 ± 13.2	66.1 ± 11.3	64.3 ± 11.4
Women, n (%)	2477 (14.7)	6245 (9.6)	8658 (11.6)	794 (5.7)	376 (4.3)	216 (3.5)
Race, n (%)						
White	12,893 (76)	51,911 (79)	61,976 (83)	12,302 (89)	7852 (90)	5474 (88)
Black	2537 (15.1)	8158 (12.5)	7262 (9.8)	920 (6.6)	446 (5.1)	438 (7.0)
Asian	348 (2.1)	712 (1.1)	764 (1.0)	103 (0.7)	34 (0.4)	31 (0.5)
Other/Mixed race	1069 (6.3)	4568 (7.0)	4344 (5.8)	558 (4.0)	346 (4.0)	277 (4.5)
Hispanic, n (%)	1136 (6.7)	4734 (7.2)	5549 (7.5)	697 (5.0)	374 (4.3)	342 (5.5)
Education, n (%)						
≤ High school/GED	3900 (23)	17,525 (27)	11,860 (16)	1768 (13)	1445 (17)	1086 (17)
Some college	6688 (40)	29,498 (45)	30,468 (41)	4797 (35)	3307 (38)	2446 (39)
≥ College degree	6259 (37)	18,326 (28)	32,018 (43)	7318 (53)	3926 (45)	2688 (43)
Income, n (%)						
< $15,000	4449 (26)	17,596 (27)	20,955 (28)	4097 (29)	2427 (28)	1749 (28)
$15,000–29,999	3629 (22)	14,380 (22)	13,534 (18)	2165 (16)	1319 (15)	1055 (17)
$30,000–44,999	3853 (23)	14,965 (23)	13,951 (19)	2280 (16)	1541 (18)	1095 (18)
≥ $45,000	4916 (29)	18,408 (28)	25,906 (35)	5341 (38)	3391 (39)	2321 (37)
Exercise days/week, n (%)						
≤ 1	9124 (55)	37,192 (57)	36,669 (49)	6115 (44)	4093 (47)	3060 (49)
2 to 4	4970 (29)	18,457 (28)	26,667 (36)	5396 (39)	3001 (35)	2067 (33)
≥ 5	2753 (16)	9700 (15)	11,010 (15)	2372 (17)	1584 (18)	1093 (18)
Smoking, n (%)						
Never	10,689 (63)	20,124 (31)	28,193 (38)	4565 (33)	2338 (27)	1602 (26)
Former	5648 (33)	40,443 (62)	40,816 (55)	8263 (60)	5554 (64)	3890 (63)
Current	510 (3.0)	4782 (7.3)	5337 (7.2)	1055 (7.6)	786 (9.1)	728 (11.7)
DASH score	21 ± 5	21 ± 5	22 ± 5	23 ± 5	22 ± 5	21 ± 5
Body mass index, kg/m^2^	29.3 ± 5.7	29.6 ± 5.8	29.2 ± 5.2	28.1 ± 4.5	28 ± 4.5	28.3 ± 4.6
HDL cholesterol, mg/dL	45 ± 13	44 ± 13	47 ± 13	51 ± 14	53 ± 15	55 ± 17
Total cholesterol, mg/dL	192 ± 42	193 ± 42	194 ± 40	196 ± 40	199 ± 38	201 ± 40
Total/HDL cholesterol ratio	4.6 ± 1.5	4.6 ± 1.6	4.4 ± 1.4	4.1 ± 1.3	4.0 ± 1.4	4.0 ± 1.3
Antihypertensive medication, n (%)	10,211 (61)	41,037 (63)	37,358 (50)	6677 (48)	4392 (51)	3106 (50)
Diabetes medication, n (%)	3677 (22)	15,091 (23)	10,058 (13)	1180 (9)	763 (9)	492 (8)
Lipid-lowering medication, n (%)	8043 (48)	32,774 (50)	30,561 (41)	5378 (39)	3564 (41)	2349 (38)
Prevalent diabetes, n (%)	5142 (31)	20,646 (32)	14,910 (20)	1932 (14)	1227 (14)	800 (13)
Prevalent hypertension, n (%)	12,798 (76)	50,429 (77)	50,558 (68)	9438 (68)	6202 (71)	4536 (73)

Data are presented as mean ± sd, unless otherwise noted

*DASH* Dietary Approaches to Stop Hypertension, *HDL* high-density lipoprotein

### Alcohol consumption and stroke

Over a median follow up of 5.2 years (Q1, Q3: 3.2, 7.1), we observed 4,339 incident stroke events (2.3%). Of these 4,339 events, 4,098 (94%) were ischemic strokes, and 241 (6%) were hemorrhagic strokes. Compared with never drinkers, light drinkers of < 1 drink/day [HR (95% CI): 0.79 (0.71, 0.89)] and moderate drinkers of 1–2 drinks/day [HR (95% CI): 0.78 (0.67, 0.92)] had a lower risk of incident stroke in Model 2 (Table 2). We did not observe a dose–response relation between higher alcohol consumption and incident stroke. Using < 1 drink/day as the reference category, we did not observe a difference between moderate consumption of 1–2 drinks/day and stroke risk. We also did not observe a substantial difference when conducting a complete-case analysis compared to the results using the imputed data (data not shown).

**Table 2:** Crude incidence rates and hazards ratios (95% CI) for incident stroke among Million Veteran Program participants who completed a lifestyle survey

	**No. Events /** **No. at risk**	**Crude incidence rate per 1,000 person-years**	**Model 1** **HR (95% CI)**^**a**^	**Model 2** **HR (95%CI)**^**b**^
Never	487/16,847	5.64	1.00 (ref.)	1.00 (ref.)
Former	1,900/65,349	5.69	1.05 (0.95, 1.17)	0.99 (0.89, 1.10)
< 1 drink/day	1,405/74,346	3.73	0.72 (0.65, 0.80)	0.79 (0.71, 0.89)
1–2 drinks/day	254/13,883	3.61	0.62 (0.53, 0.72)	0.78 (0.67, 0.92)
> 2–3 drinks/day	170/8,678	3.88	0.67 (0.56, 0.80)	0.78 (0.65, 0.94)
≥ 3 drinks/day	123/6,220	3.92	0.72 (0.59, 0.87)	0.81 (0.65, 1.00)
**Using light drinkers as reference group**				
Never			1.39 (1.25, 1.54)	1.26 (1.13, 1.41)
Former			1.46 (1.37, 1.57)	1.25 (1.16, 1.35)
< 1 drink/day			1.00 (ref.)	1.00 (ref.)
1–2 drinks/day			0.86 (0.75, 0.99)	0.99 (0.86, 1.14)
> 2–3 drinks/day			0.93 (0.79, 1.09)	0.98 (0.83, 1.16)
≥ 3 drinks/day			1.00 (0.83, 1.20)	1.02 (0.84, 1.24)

*HR* hazards ratio, *CI* confidence interval, *DASH* Dietary Approaches to Stop Hypertension

^a^Model 1 is adjusted for age and sex

^b^Model 2 is adjusted for age, sex, education, income, race, body mass index, smoking, exercise frequency, DASH score, prevalent diabetes, prevalent hypertension, lipid-lowering medication, antihypertensive medication, diabetes medication, and total/HDL cholesterol ratio

When considering incident ischemic stroke only (Table 3), we observed similar association for < 1 drink/day [HR (95% CI): 0.78 (0.70, 0.88)] and 1–2 drinks/day [HR (95% CI): 0.76 (0.65, 0.90)] compared to the overall results with combined stroke types. However, for hemorrhagic strokes, we observed no association for < 1 drink/day [HR (95% CI): 1.02 (0.60, 1.74)] and a non-statistically significant higher risk for 1–2 drinks/day [HR (95% CI): 1.29 (0.64, 2.61)] and an overall dose–response relation with increasing consumption (Table 3), though with limited precision.

**Table 3:** Multivariable adjusted hazards ratios (95% CI) for incident acute ischemic stroke and hemorrhagic stroke among Million Veteran Program participants who completed a lifestyle survey

	**No. Events /** **No. at risk**	**Acute Ischemic Stroke**	**No. Events /** **No. at risk**	**Hemorrhagic Stroke**
		**HR (95% CI)**^**a**^		**HR (95%CI)**^**a**^
Never	469/16,847	1.00 (ref.)	18/16,847	1.00 (ref.)
Former	1,787/65,349	0.97 (0.87, 1.08)	113/65,349	1.53 (0.92, 2.55)
< 1 drink/day	1,336/74,346	0.78 (0.70, 0.88)	69/74,346	1.02 (0.60, 1.74)
1–2 drinks/day	239/13,883	0.76 (0.65, 0.90)	15/13,883	1.29 (0.64, 2.61)
> 2–3 drinks/day	155/8,678	0.74 (0.61, 0.89)	15/8,678	1.81 (0.88, 3.70)
≥ 3 drinks/day	112/6,220	0.76 (0.61, 0.95)	11/6,220	1.86 (0.84, 4.10)

*HR* hazards ratio, *CI* confidence interval, *DASH* Dietary Approaches to Stop Hypertension

^a^Model is adjusted for age, sex, education, income, race, body mass index, smoking, exercise frequency, DASH score, prevalent diabetes, prevalent hypertension, lipid-lowering medication, antihypertensive medication, diabetes medication, and total/HDL cholesterol ratio

### Subgroup analyses

In age-stratified models (Table 4), among individuals age ≤ 40 years compared against never drinkers, we observed a non-statistically significant higher risk of incident stroke among those with < 1 drink/day [HR (95% CI): 1.27 (0.28, 5.73)] and 1–2 drinks/day [HR (95% CI): 2.23 (0.35, 14.16)], although the estimates were imprecise given the limited sample size and few events. Compared with never drinkers, the intake of 1–2 drinks/day was associated with a 41% lower stroke risk (95% CI: 0.35, 1.00) among individuals age 41–60 years, and a 20% lower stroke risk (95% CI: 0.67, 0.95) among those age > 60 years.

**Table 4:** Multivariable adjusted hazards ratios (95% CI) for incident stroke among Million Veteran Program participants who completed a lifestyle survey, stratified by age group

	**No. Events /** **No. at risk**	**Age ≤ 40** ***N* = 10,560**	**No. Events /** **No. at risk**	**Age 41–60** ***N* = 47,585**	**No. Events /** **No. at risk**	**Age > 60** ***N* = 127,178**
				**HR (95% CI)**^**a**^		**HR (95%CI)**^**a**^
Never	2/711	1.00 (ref.)	69/4,179	1.00 (ref.)	416/11,957	1.00 (ref.)
Former	10/2,644	1.21 (0.26, 5.70)	360/16,983	1.10 (0.84, 1.46)	1,530/45,722	0.96 (0.86, 1.08)
< 1 drink/day	18/5,795	1.27 (0.28, 5.73)	230/20,713	0.80 (0.60, 1.06)	1,157/47,838	0.79 (0.70, 0.89)
1–2 drinks/day	3/838	2.23 (0.35, 14.16)	20/2,720	0.59 (0.35, 1.00)	231/10,325	0.80 (0.67, 0.95)
> 2–3 drinks/day	0	-^b^	19/1,646	0.67 (0.38, 1.19)	151/6,749	0.78 (0.64, 0.96)
≥ 3 drinks/day	1/289	-^b^	14/1,344	0.74 (0.41, 1.33)	105/4,587	0.80 (0.64, 1.01)

*HR* hazards ratio, *CI* confidence interval, *DASH* Dietary Approaches to Stop Hypertension

^a^Model is adjusted for age, sex, education, income, race, body mass index, smoking, exercise frequency, DASH score, prevalent diabetes, prevalent hypertension, lipid-lowering medication, antihypertensive medication, diabetes medication, and total/HDL cholesterol ratio

^b^Unable to estimate HR (95% CI) from too few events

In sex-stratified models (Table 5), we observed a similar protective association to the pooled results in men for < 1 drink/day [HR (95% CI): 0.80 (0.71, 0.89)] and 1–2 drinks/day [HR (95% CI): 0.77 (0.65, 0.91)]. For women, 1–2 drinks/day was associated with a non-statistically significant higher risk of stroke [HR (95% CI): 1.30 (0.69, 2.45)].

**Table 5:** Multivariable adjusted hazards ratios (95% CI) for incident stroke among Million Veteran Program participants who completed a lifestyle survey, stratified by sex

	**No. Events /** **No. at risk**	**Men** ***N* = 166,555**	**No. Events /** **No. at risk**	**Women** ***N* = 18,764**
		**HR (95% CI)**^**a**^		**HR (95%CI)**^**a**^
Never	439/14,370	1.00 (ref.)	48/2,477	1.00 (ref.)
Former	1,781/59,104	0.99 (0.88, 1.10)	119/6,245	1.08 (0.75, 1.54)
< 1 drink/day	1,322/65,687	0.80 (0.71, 0.89)	83/8,657	0.75 (0.51, 1.10)
1–2 drinks/day	241/13,089	0.77 (0.65, 0.91)	13/794	1.30 (0.69, 2.45)
> 2–3 drinks/day	166/8,301	0.79 (0.65, 0.95)	4/376	-^b^
≥ 3 drinks/day	122/6,004	0.82 (0.66, 1.02)	1/215	-^b^

*HR* hazards ratio, *CI* confidence interval, *DASH* Dietary Approaches to Stop Hypertension

^a^Model is adjusted for age, sex, education, income, race, body mass index, smoking, exercise frequency, DASH score, prevalent diabetes, prevalent hypertension, lipid-lowering medication, antihypertensive medication, diabetes medication, and total/HDL cholesterol ratio

^b^Unable to estimate HR (95% CI) from too few events

When we stratified by the average number of hospital visits in the prior three years before baseline (Table 6), individuals with ≤ 15 hospital visits had the greatest protective association (31% reduction) with < 1 drink/day than those with > 15 visits (12–20% reduction). We observed attenuation of the association among for 1–2 drinks/day with more hospital visits [HR (95% CI): 0.80 (0.59, 1.08) for ≤ 15 visits and 0.87 (0.63, 1.19)] for ≥ 65 visits.

**Table 6:** Multivariable adjusted hazards ratios (95% CI) for incident stroke, stratified by average number of outpatient visits before baseline

	**No. Events/** **No. at risk**	**Visits** ** ≤ 15** ***N* = 47,511**	**No. Events/** **No. at risk**	**Visits** **16–33** ***N* = 45,669**	**No. Events/** **No. at risk**	**Visits** **34–64** ***N* = 45,563**	**No. Events/** **No. at risk**	**Visits** ** ≥ 65** ***N* = 46,580**
		**HR (95% CI)**^**a**^		**HR (95%CI)**^**a**^		**HR (95%CI)**^**a**^		**HR (95%CI)**^**a**^
Never	82/3,599	1.00 (ref.)	93/3,933	1.00 (ref.)	130/4,186	1.00 (ref.)	182/5,129	1.00 (ref.)
Former	253/13,119	0.86 (0.69, 1.07)	354/14,182	0.99 (0.77, 1.28)	508/16,561	1.05 (0.84, 1.30)	785/21,487	1.05 (0.88, 1.26)
< 1 drink/day	254/21,080	0.69 (0.55, 0.87)	337/19,519	0.80 (0.62, 1.04)	397/18,152	0.88 (0.70, 1.10)	417/15,595	0.88 (0.73, 1.07)
1–2 drinks/day	57/4,648	0.80 (0.59, 1.08)	62/3,899	0.79 (0.55, 1.12)	64/3,216	0.79 (0.57, 1.09)	68/2,120	0.87 (0.63, 1.19)
> 2–3 drinks/day	38/2,844	0.84 (0.60, 1.19)	37/2,426	0.58 (0.38, 0.91)	49/2,024	0.85 (0.59, 1.22)	46/1,384	0.93 (0.65, 1.32)
≥ 3 drinks/day	33/2,221	0.73 (0.48, 1.09)	34/1,710	0.94 (0.62, 1.44)	29/1,424	0.79 (0.51, 1.23)	27/865	0.95 (0.61, 1.48)

*HR* hazards ratio, *CI* confidence interval, *DASH* Dietary Approaches to Stop Hypertension

^a^Model is adjusted for age, sex, education, income, race, body mass index, smoking, exercise frequency, DASH score, prevalent diabetes, prevalent hypertension, lipid-lowering medication, antihypertensive medication, diabetes medication, and total/HDL cholesterol ratio

### Beverage preference

When restricting analyses to light and moderate drinkers to assess the association of beverage preference on incident ischemic and hemorrhagic stroke (Table 7), we did not observe a difference in ischemic stroke risk in those who preferred beer or wine compared to those having no preference. We observed a non-statistically significant higher risk of ischemic stroke among those who preferred liquor [HR (95% CI): 1.16 (0.96, 1.40)], compared to those who have no preference. We did not observe a significant difference in hemorrhagic stroke risk among those who preferred beer, wine, or liquor compared with no preference.

**Table 7:** Hazard ratios (95% CI) for beverage preference and incident ischemic and hemorrhagic stroke among Million Veteran Program participants who consume light to moderate amounts (up to 2 drinks/day) of alcohol

	**Acute Ischemic Stroke**			**Hemorrhagic Stroke**		
	**No. Events /** **No. at risk**	**Model 1** **HR (95% CI)**^**a**^	**Model 2** **HR (95%CI)**^**b**^	**No. Events /** **No. at risk**	**Model 1** **HR (95% CI)**^**a**^	**Model 2** **HR (95%CI)**^**b**^
No preference	260/16,139	1.00 (ref.)	1.00 (ref.)	24/16,139	1.00 (ref.)	1.00 (ref.)
Beer	726/38,706	1.18 (1.01, 1.39)	1.04 (0.87, 1.23)	54/38,706	0.87 (0.49, 1.55)	0.87 (0.44, 1.71)
Wine	454/23,426	1.04 (0.87, 1.24)	1.05 (0.88, 1.27)	28/23,426	0.71 (0.38, 1.35)	0.93 (0.45, 1.93)
Liquor	363/16,755	1.28 (1.07, 1.53)	1.16 (0.96, 1.40)	24/16,755	0.83 (0.42, 1.63)	0.79 (0.36, 1.75)

*HR* hazards ratio, *CI* confidence interval, *DASH* Dietary Approaches to Stop Hypertension

^a^Model 1 is adjusted for age and sex

^b^Model 2 is adjusted for age, sex, education, income, race, body mass index, smoking, exercise frequency, DASH score, prevalent diabetes, prevalent hypertension, lipid-lowering medication, antihypertensive medication, diabetes medication, and total/HDL cholesterol ratio

### Quantitative bias analysis

The quantitative bias analysis accounting for non-differential exposure misclassification demonstrated that, if we adjusted for this bias and assumed no other sources of bias existed, we would observe an even stronger protective association of moderate alcohol consumption with the risk of ischemic stroke with greater misclassification correction. The median bias-adjusted HR and 95% simulation interval (SI) was 0.47 (0.05, 0.67), compared to the original HR (95% CI) of 0.78 (0.67, 0.92). We observed some attenuation of the association [median HR (95% SI): 0.81 (0.63, 1.08)] if differential exposure misclassification with respect to comorbidity status was present in our data and adjusted for.

### Sensitivity analyses

Comparing baseline characteristics of people who did not have a Lifestyle Survey (excluded) and those who did (included), summarized in Supplemental Table 1, those who were excluded from the primary analysis were younger, had a higher proportion of women, non-White individuals, current smokers, higher BMI, and had lower income, and lower proportion of lipid-lowering medication use and prevalent hypertension. These observations suggest that, on average, those included were predominantly White, male, and had a more favorable CVD risk factor profile and higher socioeconomic status. There was a similar distribution of alcohol consumption between the two groups, but those who were excluded had a slightly higher proportion of < 1 drink/day, while those who were included were more likely to consume 1–2 drinks/day. We observed 3,979 incident stroke events (1.6%) in those who were excluded and 4,634 incident stroke events (2.3%) in those who were included.

In analyses using the AUDIT-C defined alcohol consumption (*N* = 448,495, Supplemental Table 2) to compare the association of moderate drinking with stroke risk between those who were excluded (*N* = 250,445) vs. included (*N* = 198,050). We observed similar estimates of < 1 drink/day [HR (95% CI): 0.88 (0.82, 0.95) among excluded vs. 0.86 (0.81, 0.92) among included] and 1–2 drinks/day [HR (95% CI): 0.83 (0.71, 0.96) among excluded vs. 0.80 (0.72, 0.89) among included]. Using < 1 drink/day as the reference group, never drinkers had a slightly higher risk of stroke (13–16%) and was similar between those included and excluded. Lastly, we additionally adjusted for prevalent depression as it is associated with alcohol consumption and stroke risk but did not observe a change in the HR (95% CI) among those who consume moderate amounts (data not shown).

## Discussion

We observed that moderate alcohol consumption was associated with a 22% lower risk of total stroke compared to never drinking, and consumption of 1–2 drinks/day had a similar association as consuming < 1 drink/day. Previous studies assessing this association in multi-ethnic cohorts have found similar effect sizes of moderate alcohol consumption and stroke risk [7], even up to 4 drinks/day [28]. A recent large international study observed a 29% lower odds of stroke among moderate drinkers in Western Europe and North America demonstrating that the protective association is consistently observed using observational data; however, other regions observed a higher odds of stroke with moderate consumption [29]. When restricting to light and moderate drinkers, we did not observe a difference in ischemic stroke risk between those who preferred beer or wine compared to those who consumed all beverage types and a non-statistically significant higher risk of ischemic stroke in those who preferred liquor. Previous studies have primarily found greater protective benefits of red wine consumption [30, 31]. However, we only examined the effect of dominant beverage type, defined as consuming ≥ 50% of a particular beverage of an individual’s total alcohol consumption. It is possible that even some consumption of red wine will confer a health benefit.

When examining stroke type separately, we observed a 22–24% lower risk of ischemic stroke with the consumption of up to two drinks/day. We observed no association for < 1 drink/day with hemorrhagic strokes. We observed with limited precision a dose–response relation of increasing alcohol consumption with greater hemorrhagic stroke risk. The observed difference in association between ischemic versus hemorrhagic stroke outcomes is consistent with previous meta-analyses assessing stroke type that reported a protective association for ischemic stroke but no association or higher risk for hemorrhagic stroke [5, 32, 33]. The increase in hemorrhagic stroke risk may be due to the effect of antiplatelet aggregation with alcohol consumption thereby leading to a higher propensity of bleeding, or alcohol consumption increasing the risk of hypertension, as noted by previous studies [34–36].

There has also been conflicting evidence about the mechanism by which alcohol consumption provides protective benefits against CVD risk through platelet aggregation, reducing inflammation, and the increase of HDL cholesterol. We observed increasing mean HDL cholesterol with greater alcohol consumption in our sample. Adjustment for HDL did not substantially impact the effect size (data not shown), which was also observed in studies of coronary heart disease with additional HDL adjustment [37]. Despite some Mendelian randomization studies finding alcohol consumption is associated with increased HDL; the latter is likely not a major contributor to the potential benefits of alcohol consumption [38, 39].

### Impact of bias on observed results

There is still an ongoing debate whether the observed U-shaped relation of alcohol consumption and various health outcomes is a true effect or whether specific biases can explain the observed protective association of alcohol intake. Confounding related to the decision to consume alcohol, selection bias related to willingness to participate in research studies, the timing of study enrollment, and reverse causation (prevalent health conditions that affect current alcohol consumption) all have been noted to be potential sources of bias in studies of alcohol consumption and mortality [16]. In our sensitivity analyses to examine for indications of selection bias, we observed differences in baseline characteristics between those included and excluded from our primary analysis. However, we observed a similar protective association of moderate consumption, 17% reduction among those excluded and 20% reduction among those included, on stroke risk using the AUDIT-C defined exposure. We also examined if the association could be due to “healthy survivor” bias, given that our sample was primarily comprised of older individuals, and younger individuals may be more susceptible to alcohol-related death. We stratified our analysis by age group and observed a higher risk of stroke in those < 40 years of age using the survey-based exposure; however, the estimates were very imprecise compared to those ≥ 40. When stratifying by age group using the AUDIT-C measure, which provided a larger sample of younger individuals, we observed no association of moderate consumption in younger individuals compared to older individuals (data not shown). Lastly, we conducted analyses stratified by the prior number of hospital visits in the three years before baseline to assess if we observed similar protective effects among those who have worse health status. We observed a HR (95% CI) of 0.80 (0.59, 1.08) in those with ≤ 15 visits and 0.87 (0.63, 1.19) in those with ≥ 65 visits among moderate consumers compared to never drinking. Our findings provide some evidence that healthy user bias related to moderate alcohol consumption may be affecting the observed protective association.

### Limitations

Our study had limitations. We used a single self-reported measure of alcohol consumption that was collected from the survey or the AUDIT-C and may not accurately represent an individual’s history of alcohol consumption, especially true “never drinkers.” Total alcohol consumption was derived from the survey using separate questions for beer, wine, and liquor consumption and may be subject to exposure misclassification. Due to the prospective nature of the study design, any misclassification is likely non-differential. We would have expected a bias towards the null, as our quantitative bias analysis demonstrated. Furthermore, our assessment of exposure misclassification with respect to comorbidity status did not demonstrate a substantial impact on the observed protective effect. However, the bias analysis only assessed the impact of these sources of bias individually, so we cannot determine the impact when considering all sources acting together. We may not have adequately controlled for all confounding factors as the data were obtained through either self-report or the EHR, which has been noted to be a source for the observed protective association of alcohol consumption [40]. Incident stroke was defined using ICD-9 and ICD-10 codes found in the EHR, and some events may have been missed. However, we would not expect differential outcome ascertainment with respect to alcohol consumption due to the prospective nature of our study. We also did not find a difference in the number of imaging visits between those who were never or moderate drinkers that may have resulted in the better ascertainment of stroke cases in the exposed vs. unexposed. Furthermore, Veterans who seek care in community settings may not have been included in this study which is another potential source of selection bias. We did not have a large enough sample of women and younger individuals to make strong inferences about the association of alcohol consumption in these sub-groups. Despite these limitations, our large sample size allowed sensitivity analyses and us to conduct several stratified, and had access to two alcohol consumption variables to assess the impact of selection bias and misclassification.

### Conclusion

In conclusion, moderate alcohol consumption was associated with a lower risk of ischemic stroke but not hemorrhagic stroke in our data with no substantial difference among those who preferred a particular beverage type. However, the protective association was attenuated in individuals who have lower health status suggesting that healthy user bias may have affected the observed results.

### Supplementary Information

**Additional file 1:** **Supplemental Table** **1.** Baseline characteristics comparing all eligible 448,495 Million Veteran Program participants, participants without a lifestyle survey, and participants with a lifestyle survey. **Supplemental Table 2.** Multivariable adjusted hazards ratio (95% CI) for incident stroke using alcohol consumption from the AUDIT-C, comparing survey responders and non-survey responders.

## Data Availability

The datasets generated and/or analyzed during the current study are not publicly available due to containing protected health information. There are regulatory restrictions on sharing patient-level data used in these analyses, even if it is de-identified. Nicole Usher (Nicole.Usher@va.gov) is the point of contact for Million Veteran Program data access.

## Abbreviations

AUDIT-C: Alcohol Use Disorder Test-Concise
BMI: Body mass index
CVD: Cardiovascular disease
DASH: Dietary approaches to Stop Hypertension
EHR: Electronic health record
HDL: High density lipoprotein
ICD: International Classification of Diseases
MVP: Million Veteran Program
NPV: Negative predictive value
PPV: Positive predictive value
VA: Veterans Affairs
