# Supplementary material for: Moderate alcohol consumption on the risk of stroke in the Million Veteran Program
Source: BMC Public Health. 2023 Dec 12;23:2485. doi: 10.1186/s12889-023-17377-x (PMC10714616; doi:10.1186/s12889-023-17377-x)
Supplement: Supplementary file 1 — Additional file 1: Supplemental Table 1. Baseline characteristics comparing all eligible 448,495 Million Veteran Program participants, participants without a lifestyle survey, and participants with a lifestyle survey. Supplemental Table 2. Multivariable adjusted hazards ratio (95% CI) for incident stroke using alcohol consumption from the AUDIT-C, comparing survey responders and non-survey responders. [file 12889_2023_17377_MOESM1_ESM.docx]

**Supplemental Table** **1**. Baseline characteristics comparing all eligible 448,495 Million Veteran Program participants, participants without a lifestyle survey, and participants with a lifestyle survey

|  | **All eligible participants** | **No Lifestyle** | **Lifestyle** |
| --- | --- | --- | --- |
| **Characteristic** | **N=448,495** | **N=250,445** | **N=198,050** |
| Age, years | 58.6 ± 15.1 | 54.5 ± 15.6 | 63.8 ± 12.6 |
| Women, n (%) | 56,830 (13) | 35,698 (14) | 21,132 (11) |
| Race, n (%) |  |  |  |
| White | 325,443 (75) | 164,084 (68) | 161,359 (82) |
| Black | 78,161 (18) | 57,189 (24) | 20,972 (11) |
| Asian | 7,162 (1.6) | 5,019 (2.1) | 2,143 (1.1) |
| Other/Mixed race | 25,571 (5.9) | 13,590 (5.7) | 11,981 (6.1) |
| Hispanic, n (%) | 40,951 (9.2) | 27,264 (11.1) | 13,687 (6.9) |
| Income, n (%) |  |  |  |
| <$15,000 | 128,732 (30) | 75,337 (32) | 53,395 (28) |
| $15,000-29,999 | 96,244 (22) | 58,568 (25) | 37,676 (20) |
| $30,000-44,999 | 88,422 (21) | 49,193 (21) | 39,229 (20) |
| ≥$45,000 | 117,318 (27) | 54,651 (23) | 62,667 (33) |
| Alcohol consumption (AUDIT-C), n (%) |  |  |  |
| 0 drinks/day | 184,736 (41) | 104,155 (42) | 80,581 (41) |
| <1 drink/day | 214,846 (48) | 122,997 (49) | 91,849 (46) |
| 1-2 drinks/day | 36,944 (8.2) | 16,902 (6.8) | 20,042 (10) |
| >2-3 drinks/day | 7,152 (1.6) | 3,617 (1.4) | 3,535 (1.8) |
| ≥3 drinks/day | 4817 (1.1) | 2774 (1.1) | 2043 (1.0) |
| Smoking, n (%) |  |  |  |
| Never | 151,192 (37) | 84,931 (37) | 66,261 (37) |
| Former | 219,517 (53) | 117,298 (51) | 102,219 (56) |
| Current | 40,860 (10) | 27,941 (12) | 12,919 (7) |
| Body mass index, kg/m^2^ | 29.8 ± 5.7 | 30.3 ± 5.9 | 29.2 ± 5.4 |
| Diastolic blood pressure, mm Hg | 78 ± 10 | 78 ± 10 | 77 ± 10 |
| Systolic blood pressure, mm Hg | 130 ± 16 | 130 ± 16 | 131 ± 16 |
| A1C, % | 6.0 ± 1.3 | 6.0 ± 1.4 | 6.0 ± 1.2 |
| HDL cholesterol, mg/dL | 46 ± 14 | 46 ± 14 | 47 ± 14 |
| Total cholesterol, mg/dL | 194 ± 42 | 194 ± 42 | 194 ± 41 |
| Antihypertensive medication, n (%) | 246,265 (55) | 136,325 (54) | 109,940 (56) |
| Diabetes medication, n (%) | 79,063 (18) | 45,249 (18) | 33,814 (17) |
| Lipid-lowering medication, n (%) | 186,450 (42) | 97,903 (39) | 88,547 (45) |
| Prevalent diabetes, n (%) | 107,296 (24) | 59,113 (24) | 48,183 (24) |
| Prevalent hypertension, n (%) | 312,693 (70) | 169,552 (68) | 143,141 (72) |

HDL, high-density lipoprotein

Data are presented as mean ± sd, unless otherwise noted.

**Supplemental Table 2**. Multivariable adjusted hazards ratio (95% CI) for incident stroke using alcohol consumption from the AUDIT-C, comparing survey responders and non-survey responders

|  | **No. Events /**  **No. at risk** | **All eligible participants**  **N=448,495** | **No. Events /**  **No. at risk** | **No Lifestyle survey (excluded)**  **N=250,445** | | **No. Events /**  **No. at risk** | | **With Lifestyle survey**  **(included)**  **N=198,050** |
| --- | --- | --- | --- | --- | --- | --- | --- | --- |
|  |  | **HR (95% CI)^1^** |  | **HR (95%CI)^1^** | |  | | **HR (95% CI)^1^** |
| Never | 4,316/184,736 | 1.00 (ref.) | 2,090/104,155 | 1.00 (ref.) | | 2,226/80,851 | | 1.00 (ref.) |
| <1 drink/day | 3,440/214,846 | 0.86 (0.82, 0.91) | 1,572/122,997 | 0.88 (0.82, 0.95) | | 1,868/91,849 | | 0.86 (0.81, 0.92) |
| 1-2 drinks/day | 659/36,944 | 0.79 (0.73, 0.87) | 236/16,902 | 0.83 (0.71, 0.96) | | 423/20,042 | | 0.80 (0.72, 0.89) |
| >2-3 drinks/day | 124/7,152 | 0.80 (0.65, 0.97) | 48/3,617 | 0.87 (0.64, 1.18) | | 76/3,535 | | 0.77 (0.60, 1.00) |
| ≥3 drinks/day | 74/4,817 | 0.90 (0.69, 1.17) | 33/2,774 | 0.81 (0.55, 1.21) | | 41/2,043 | | 0.99 (0.70, 1.40) |
| **Using light drinkers as reference group** | | | | |  | |  |  |
| Never |  | 1.16 (1.1, 1.22) |  | 1.13 (1.06, 1.22) | |  | | 1.16 (1.09, 1.24) |
| <1 drink/day |  | 1.00 (ref.) |  | 1.00 (ref.) | |  | | 1.00 (ref.) |
| 1-2 drinks/day |  | 0.92 (0.84, 1.00) |  | 0.94 (0.81, 1.09) | |  | | 0.93 (0.83, 1.04) |
| >2-3 drinks/day |  | 0.92 (0.76, 1.12) |  | 0.98 (0.72, 1.34) | |  | | 0.90 (0.69, 1.16) |
| ≥3 drinks/day |  | 1.04 (0.80, 1.35) |  | 0.92 (0.62, 1.37) | |  | | 1.15 (0.82, 1.63) |

AUDIT-C, Alcohol use disorders Identification Test; HR, hazards ratio; CI, confidence interval

^1^Model is adjusted for age, sex, income, race, body mass index, smoking, prevalent diabetes, prevalent hypertension, lipid-lowering medication, antihypertensive medication, diabetes medication, and total/HDL cholesterol ratio
